# Multisystem Inflammatory Syndrome in Adult after First Dose of mRNA Vaccine

**DOI:** 10.3201/eid2804.212585

**Published:** 2022-04

**Authors:** Yusuke Miyazato, Kei Yamamoto, Gen Yamada, Shuji Kubota, Masahiro Ishikane, Masaya Sugiyama, Mikako Ueno, Akihiro Matsunaga, Tohru Miyoshi-Akiyama, Yukihito Ishizaka, Norio Ohmagari

**Affiliations:** National Center for Global Health and Medicine, Tokyo, Japan (Y. Miyazato, K. Yamamoto, G. Yamada, S. Kubota, M. Ishikane, M. Ueno, A. Matsunaga, T. Miyoshi-Akiyama, Y. Ishizaka, N. Ohmagari);; National Center for Global Health and Medicine, Chiba, Japan (M. Sugiyama)

**Keywords:** COVID-19, coronavirus disease, SARS-CoV-2, severe acute respiratory syndrome coronavirus 2, vaccination, respiratory failure, myocardial injury, MIS-A, hyperinflammatory state, cytokine profile, anti-spike antibody, anti-nucleocapsid antibody, multisystem inflammatory syndrome, Japan

## Abstract

A 32-year-old man in Japan experienced respiratory failure after receiving the first dose of coronavirus disease (COVID-19) vaccine. He was treated with noninvasive ventilation and corticosteroids. Serologic test results suggested previous COVID-19; therefore, he received a diagnosis of multisystem inflammatory syndrome. COVID-19 vaccination could be a trigger for this condition.

A 32-year-old man from France living in Tokyo was admitted to the National Center for Global Health and Medicine after experiencing shortness of breath and fever. He had received the first dose of the BNT162b2 (Pfizer-BioNTech, https://www.pfizer.com) vaccine 5 days before admission. After vaccination, he experienced a fever, systemic joint pain, nausea, and vomiting. The patient sought care because of these persistent symptoms. 

At admission, the patient was experiencing dyspnea as well as chest and back pain that worsened during inhalation. The patient was obese (body mass index 42.1 kg/m^2^). He had no history of smoking, illegal drug use, or international travel. When he received the vaccine, Japan was experiencing its largest coronavirus disease (COVID-19) surge, but he had no known exposure to patients with COVID-19. At admission, he had a body temperature of 38.1°C and peripheral oxygen saturation (SpO_2_) of 95% on room air (Table). He had no notable jugular venous dilation, chest crackles, peripheral edema, or rashes.

**Table:** Clinical features and laboratory results of a patient who experienced multisystem inflammatory syndrome in an adult after a coronavirus vaccination, Japan, 2021

Characteristic	Hospital day 1	Hospital day 2	Hospital day 3	Hospital day 5	Day of discharge (day 8)	1 month after discharge	Reference range
Clinical features							
Maximum body temperature, °C	38.1	39.1	36.8	36.8	36.8	36.0	NA
Maximum respiratory rate, breaths/min	20	35	26	22	18	NA	NA
Maximum heart rate, bpm	126	128	120	111	100	NA	NA
Minimum blood pressure, mm Hg	102/81	105/85	113/88	141/85	135/85	NA	NA
Laboratory results							
SARS-CoV-2 real-time PCR	Negative	NA	NA	NA	NA	NA	Negative
SARS-CoV-2 spike IgG	Positive	NA	NA	NA	NA	Positive (day 19)	Negative
SARS-CoV-2 nucleocapsid IgG	Positive	NA	NA	NA	NA	NA	Negative
Leukocytes, cells/µL	12,790	16,330	14,280	13,380	17,680	4,780	3,300–8,600
Platelets, × 10^3^/µL	166	217	240	294	341	208	158–348
Creatinine, mg/dL	1.02	1.14	1.26	1.09	0.95	1.07	0.65–1.07
LDH, U/L	210	228	225	227	214	213	124–222
Troponin I, ng/mL	0.371	1.102	1.306	0.295	0.094	NA	0–0.026
BNP, pg/mL	129.3	409.5	NA	NA	68.0	NA	0–18.4
CRP, mg/dL	30.73	35.82	33.34	10.35	1.98	0.08	0–0.14
Ferritin, ng/mL	880.0	NA	NA	NA	NA	NA	21–282
ESR, mm/h	NA	NA	NA	NA	49	NA	2–10
IL-6, pg/mL	NA	NA	NA	99.29	0 (day 9)	0 (day 44)	0
Treatment							
Oxygen delivery devices	Nasal cannula	NIV	NIV	Nasal cannula	None	None	NA
Corticosteroids	None	mPSL 125 mg/d (1 mg/kg/d) IV	mPSL 125 mg/d (1 mg/kg/d) IV	PSL 60 mg orally	None	None	NA
Diuretics	Furosemide 20 mg orally	Furosemide 40 mg IV	Furosemide 40 mg IV	Furosemide 20 mg orally	None	None	NA
Antimicrobial drugs	LVFX 500 mg orally	LVFX 500 mg orally	None	None	None	None	NA

*BNP, brain natriuretic peptide; CRP, C-reactive protein; ESR, erythrocyte sedimentation rate; IL-6, interleukin-6; IV, intravenous; IgG, immunoglobulin G; LDH, lactate dehydrogenase; LVFX, levofloxacin; mPSL, methylprednisolone; NA, not applicable; NIV, noninvasive ventilation; PSL, predonisolone; SARS-CoV-2, severe acute respiratory syndrome coronavirus 2.

Laboratory test results showed an elevated inflammatory response and cardiac enzymes (Table). Chest computed tomography (CT) showed smooth interlobular septal thickening, mixed lesions with ground-glass opacitie, and infiltrates in the bilateral lower lobes (Figure, panel A). Electrocardiography showed slight ST segment elevations in leads I, aVL, V1, and V2. Echocardiography showed no pericardial effusion, myocardial edema, or decreased wall motion. Real-time PCR results were negative for severe acute respiratory syndrome coronavirus 2 (SARS-CoV-2). Loop-mediated isothermal amplification did not detect *Legionella pneumophila.* We used FilmArray version 1.3 (bioMérieux, https://www. biomerieux.com) to conduct a respiratory panel on respiratory specimens and a meningitis/encephalitis panel on serum specimens to detect herpesvirus, enterovirus, and cytomegalovirus; results of both panels were negative.

**Figure:**
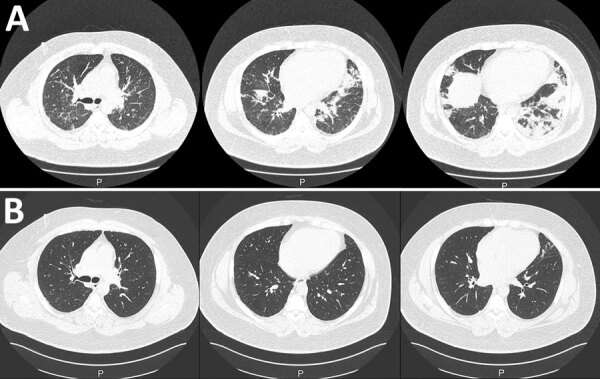
Chest computed tomography (CT) images of a male patient in Japan who was hospitalized with multisystem inflammatory syndrome. A) CT performed at hospital admission revealed infiltration in bilateral lower lobes. B) Chest CT performed 1 month after discharge revealed that most of these lesions had resolved.

One day after admission, the patient’s dyspnea and hypoxemia worsened, and he experienced profuse cold sweats. His SpO_2_ dropped to 90% despite 10 L/min of oxygen supply. We suspected severe respiratory failure resulting from COVID-19 vaccine–related systemic inflammation and congestive heart failure. Therefore, we treated the patient with intravenous methylprednisolone at a dose of 1 mg/kg/day (125 mg/d) and with diuretics and noninvasive ventilation (NIV). The next day, his symptoms and hypoxemia greatly improved. He tapered off both treatments; he no longer needed ventilation 2 days after treatment and completed the course of steroids by the day of discharge, 7 days after admission. One month after discharge, CT was performed to confirm the improvement in the lung lesions (Figure, panel B).

Testing showed that SARS-CoV-2 spike IgG and neutralizing activities were significantly elevated 5 days and 23 days after the first COVID-19 vaccination dose had been administered (Appendix Figure 1). Moreover, SARS-CoV-2 nucleocapsid IgG in the serum was positive 5 days after COVID-19 vaccination. On the basis of these findings, we hypothesize that the patient had an asymptomatic or mild SARS-CoV-2 infection before vaccination. After his discharge, we measured a panel of 67 cytokines and chemokines from the patient and 3 healthy controls for comparison (Appendix Table, Figure 2).

This case emphasized 2 clinical issues. First, severe respiratory failure can occur after COVID-19 vaccination, and steroids effectively alleviated this complication. Second, multisystem inflammatory syndrome in adults (MIS-A) can occur after COVID-19 vaccination in a previously infected patient and can manifest as respiratory distress. In cases of respiratory failure after the vaccination, a previous SARS-CoV-2 infection should be considered.

Postvaccination myocarditis has been reported as more common in male than female patients (*1*). Bozkurt et al. described mild cases (*1*); however, severe cases have also been reported (*2*). Although our patient’s myocardial damage was not severe, we suspected myocarditis based on his elevated troponin I level after COVID-19 vaccination. Vaccine-related myocarditis typically develops after the second vaccination, but it has been reported after the first vaccination of patients who had COVID-19 previously (*1*). Therefore, we considered the possibility of myocarditis after the first vaccination in this patient, because his serology results suggested a history of COVID-19. Moreover, his respiratory failure, severe inflammation, and serologic test results strongly suggesting a history of COVID-19 led us to suspect MIS-A, as reported by Morris et al. (*3*). Although the association between the COVID-19 vaccine and MIS-A development is unclear (*4*), the patient in our case fulfilled the clinical criteria of severe cardiac illness, hypotension, vomiting, and fever. In addition, his laboratory results showed elevated C-reactive protein levels, ferritin levels, interleukin-6 levels, and erythrocyte sedimentation rate. He also exhibited serologic positivity for SARS-CoV-2. These findings were consistent with the definition of MIS-A (*5*). This case showed that vaccination was a possible trigger of MIS-A in a patient who had a history of COVID-19.

The treatment for postvaccination myocarditis and MIS-A has not been standardized. As demonstrated in our case, immunosuppressive therapy, particularly corticosteroids, improved the prognosis. Intravenous immunoglobulin, anakinra, and infliximab have been used to treat multisystem inflammatory syndrome in children (*6*,*7*); a previous case report documented their role in treating MIS-A (*8*). 

Appendix Additional information about multisystem inflammatory syndrome in an adult after first dose of mRNA vaccine, Japan, 2021.
