# Supplementary material for: Multisystem Inflammatory Syndrome in Adult after First Dose of mRNA Vaccine
Source: Emerg Infect Dis. 2022 Apr;28(4):870–2. doi: 10.3201/eid2804.212585 (PMC8962876; doi:10.3201/eid2804.212585)
Supplement: Appendix — Additional information about multisystem inflammatory syndrome in an adult after first dose of mRNA vaccine, Japan, 2021. [file 21-2585-Techapp-s1.pdf]

Article DOI: https://doi.org/10.3201/eid2804.212585

Multisystem Inflammatory Syndrome in Adult after First Dose of mRNA Vaccine

Appendix

Additional Methods

After discharge, we measured a panel of 67 cytokines and chemokines from the patient and 3 healthy controls using Bio-plex Pro Human Chemokine assays (Bio-Rad, https://www.bio-rad.com) (Appendix Table, Appendix Figure 2) to investigate abnormal responses induced by coronavirus disease. The patient's BCA-1/CXCL13, HGF, IL-5, IL-6, IL-8/CXCL8, IL-13, IL-15, IL-16, IP-10/CXCL10, MIF, MPIF-1/CCL23, PDGF-BB, and VEGF levels were elevated before steroid administration compared to healthy controls and became almost normal during the recovery period.

Among the cytokines known to be elevated in the cytokine storm, IL-1, IL-2, IL-6, IL-7, IL-12, IL-17, IL-18 TNF-α, MCSF, G-CSF, CXCL-10/IP-10, CCL-3, CCL-5, IFN-γ, and MCP-1, only IL-6 and CXCL-10/IP-10 were elevated in the acute phase (*1*). We believe that the elevation of IL-5, IL-16, MIF, and VEGF are also characteristic of this case. IL-5, IL-16, and MIF have been reported to be elevated in myocardial injury (*2*–*4*), and MIF and VEGF have been suggested to be associated with vasculitis (*5*,*6*), which may be related to the pathogenesis of this case.

References

1. Mariappan V, Manoharan PS, R P, Shanmugam L, Rao SR, Pillai AB. Potential biomarkers for the early prediction of SARS-COV-2 disease outcome. Microb Pathog. 2021;158:105057. PubMed https://doi.org/10.1016/j.micpath.2021.105057

2. Song T, Jones DM, Homsi Y. Therapeutic effect of anti-IL-5 on eosinophilic myocarditis with large pericardial effusion. BMJ Case Rep. 2017;2017:bcr-2016-218992. PubMed https://doi.org/10.1136/bcr-2016-218992

3. Zhang J, Yang Z, Liang Z, Wang M, Hu C, Chang C, et al. Anti-interleukin-16-neutralizing antibody attenuates cardiac inflammation and protects against cardiac injury in doxorubicin-treated mice. Mediators Inflamm. 2021;2021:6611085. PubMed https://doi.org/10.1155/2021/6611085

4. Fan F, Fang L, Moore XL, Xie X, Du XJ, White DA, et al. Plasma macrophage migration inhibitor factor is elevated in response to myocardial ischemia. J Am Heart Assoc. 2016;5:e003128. PubMed https://doi.org/10.1161/JAHA.115.003128

5. Wakabayashi K, Otsuka K, Sato M, Takahashi R, Odai T, Isozaki T, et al. Elevated serum levels of macrophage migration inhibitory factor and their significant correlation with rheumatoid vasculitis disease activity. Mod Rheumatol. 2012;22:59–65. PubMed https://doi.org/10.3109/s10165-011-0466-z

6. Yücel Ç, Sertoğlu E, Fırat Oğuz E, Hayran Y, Omma A, Özgürtaş T. Serum VEGF-A and VEGFR-1 levels in patients with adult immunoglobulin A vasculitis. Int J Rheum Dis. 2021;24:789–94. PubMed https://doi.org/10.1111/1756-185X.14115

Appendix Table. Profile of 67 cytokines and chemokines from a patient with multisystem inflammatory syndrome in an adult and 3 healthy persons who had received 2 doses of mRNA vaccine for coronavirus disease

Cytokines and	Patient					Controls		
chemokines	Day 5	Day 6	Day 9	Day 23	Day 44	N1	N2	N3
BCA-1/CXCL13	91.93	93.02	33.63	27.79	33.04	26.81	27.16	24.96
HGF	860.57	916.26	513.33	420.42	462.91	328.58	242.75	187.56
IL-5	45.35	48.35	0.00	0.00	0.00	0.00	0.00	0.00
IL-6	99.29	79.43	0.00	0.00	0.00	0.00	0.00	0.00
IL-8/CXCL8	23.01	21.97	10.25	12.13	14.56	9.03	10.68	7.85
IL-13	1.73	1.93	0.00	0.00	0.00	0.00	0.00	0.00
IL-15	573.25	551.10	0.00	0.00	0.00	0.00	0.00	0.00
IL-16	251.27	192.02	86.50	108.90	121.02	91.20	81.37	73.20
IP-10/CXCL10	923.37	836.39	103.41	92.82	152.13	197.93	97.95	150.18
MIF	21,666.34	45,596.47	17,066.50	24,660.14	2,433.82	1,568.66	1,421.55	2,559.85
MPIF-1/CCL23	917.16	1,077.75	317.36	249.18	185.88	168.91	67.99	291.75
PDGF-BB	7,437.96	5,179.37	3,261.03	2,455.29	2,895.53	4,077.71	1,628.17	2,180.10
VEGF	227.94	271.61	0.00	0.00	0.00	0.00	0.00	0.00
b-NGF	30.04	30.13	6.32	7.72	3.19	0.00	0.00	0.00
GCP-2/CXCL6	160.60	127.54	234.13	194.95	373.96	29.53	27.86	37.87
G-CSF	808.20	572.19	1,101.25	1,189.84	3,463.18	116.85	166.68	122.80
Gro-b/CXCL2	613.63	558.26	874.69	747.56	1,276.49	199.66	315.38	296.14
IFN-a2	9.54	7.06	4.75	5.93	8.34	0.00	0.00	0.00
IL-1a	38.68	31.24	22.65	25.09	21.90	2.01	2.01	0.19
IL-1ra	869.98	878.00	130.95	172.08	309.16	44.71	0.00	0.00
IL-2Ra	118.62	116.42	79.87	80.98	108.86	50.41	40.80	46.40
IL-12(p40)	180.16	128.66	124.88	113.61	181.62	65.73	35.30	35.30
IL-17	12.88	8.42	2.49	4.62	9.87	0.00	0.00	0.00
IL-18	113.03	93.66	53.68	56.97	60.35	20.16	35.33	19.20
I-TAC/CXCL11	128.64	130.71	258.81	158.94	79.01	22.94	17.68	30.81
M-CSF	39.22	29.75	26.71	27.35	22.10	7.17	11.19	10.18
MIP-1a/CCL3	267.53	194.33	378.72	435.93	1554.19	4.76	7.27	4.76
MIP-1d/CCL15	5,363.92	5,592.40	3,610.77	3,456.25	4,886.33	2,536.68	2,686.14	1,751.91
6Ckine/CCL21	26,044.85	39,882.28	17,327.50	19,974.72	59,927.96	23,972.90	25,506.51	21,218.49
ENA-78/CXCL5	1,212.70	977.62	942.50	695.23	3,312.40	825.19	776.21	764.77

Cytokines and chemokines	Patient					Controls		
	Day 5	Day 6	Day 9	Day 23	Day 44	N1	N2	N3
Eotaxin-2/CCL24	610.70	733.43	1160.59	968.08	976.64	428.15	526.03	332.06
Gro-a/CXCL1	365.64	347.95	376.18	393.44	921.62	218.10	174.88	182.84
LIF	77.01	70.30	61.98	66.18	112.24	50.08	30.82	52.39
MCP-3/CCL7	0.00	0.00	0.00	0.00	42.59	0.00	0.00	0.00
MCP-4/CCL13	66.89	60.32	59.43	57.52	134.17	60.58	66.21	53.50
BasicFGF	51.09	40.12	33.58	37.90	64.48	43.51	38.95	47.77
CTACK/CCL27	1103.94	746.55	438.94	502.99	873.03	878.66	759.24	1123.30
Eotaxin/CCL11	47.07	53.18	34.86	37.49	59.50	47.78	67.31	63.64
Eotaxin-3/CCL26	83.57	81.38	79.49	70.25	113.99	94.96	100.01	104.82
Fractalkine/CX3CL1	126.98	112.19	154.47	149.99	222.58	133.50	119.69	181.95
GM-CSF	25.22	24.23	25.16	22.18	33.92	21.10	24.61	24.70
I-309/CCL1	32.89	30.92	29.11	31.80	61.86	43.29	44.17	54.29
IFN-g	27.31	26.81	30.19	26.10	37.50	30.88	24.68	25.84
IL-1b	6.29	6.76	5.89	6.09	8.62	6.68	7.35	7.51
IL-2	0.00	0.00	0.00	0.00	14.05	7.31	5.32	8.90
IL-3	0.01	0.00	0.00	0.00	0.00	0.00	0.00	0.00
IL-4	81.83	75.30	83.59	80.30	107.60	68.40	70.87	80.52
IL-7	9.74	12.49	0.00	0.00	7.35	7.35	10.87	0.07
IL-9	208.11	228.20	226.58	216.36	327.58	297.72	281.09	311.00
IL-10	8.16	8.79	6.89	6.72	10.54	8.03	6.42	8.40
IL-12(p70)	0.00	0.00	0.00	0.00	0.00	0.00	0.00	0.00
MCP-1/CCL2	53.91	64.66	46.43	52.05	79.06	60.63	69.33	62.39
MCP-2/CCL8	53.48	50.60	26.42	31.40	71.61	101.63	54.37	55.22
MDC/CCL22	792.45	701.66	474.66	665.63	928.56	541.04	662.62	620.09
MIG/CXCL9	56.05	47.46	16.67	16.96	41.42	30.58	34.09	34.39
MIP-1b	224.02	225.72	217.14	221.23	244.88	221.81	196.70	209.23
MIP-3a/CCL20	0.00	0.00	0.00	0.00	0.00	0.00	0.00	0.00
MIP-3b/CCL19	138.39	135.39	0.00	15.78	204.26	127.83	107.47	147.32
RANTES	9,363.29	8,820.77	6,618.98	6,886.85	8,146.81	7,986.42	6,914.73	7,588.66
SCF	75.83	64.30	46.75	59.98	95.11	75.33	68.48	99.40
SCGF-b	141,179.79	108,130.23	151,784.32	166,409.90	174,281.40	113,842.98	149,646.34	157,163.45

Cytokines and chemokines	Patient					Controls		
	Day 5	Day 6	Day 9	Day 23	Day 44	N1	N2	N3
SCYB16/CXCL16	863.50	958.28	630.81	624.50	709.56	553.42	573.47	542.07
SDF-1a+b/CXCL12	2,704.63	2514.82	1,580.83	1,512.64	2,530.59	2,178.87	1,762.36	2,021.26
TECK/CCL25	1,045.18	1,147.02	824.00	902.54	1,289.05	1,052.93	1,019.89	997.06
TNF-a	130.18	126.50	99.40	95.10	167.69	117.97	122.05	134.08
TNF-b	279.37	265.23	274.34	306.91	339.22	287.75	320.74	304.75
TRAIL	13.45	13.23	12.33	12.61	18.53	18.43	13.00	15.06

*Values are expressed in pg/mL. Values below the detection sensitivity are indicated as 0.00.

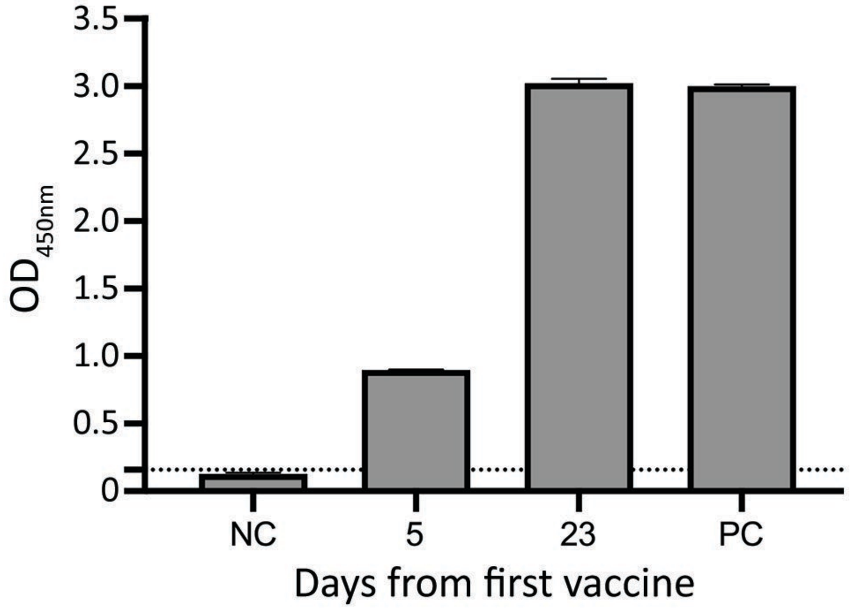

Appendix Figure 1. Severe acute respiratory syndrome coronavirus 2 anti-spike (full-length) antibody titers from a patient with multisystem inflammatory syndrome in an adult. Antibody induction was observed within 5 days after vaccination. High antibody titers were obtained 23 days after vaccination. Dotted line indicates the positivity criterion, which was based on the negative control mean + 3 SD. NC, negative control; PC, positive control.

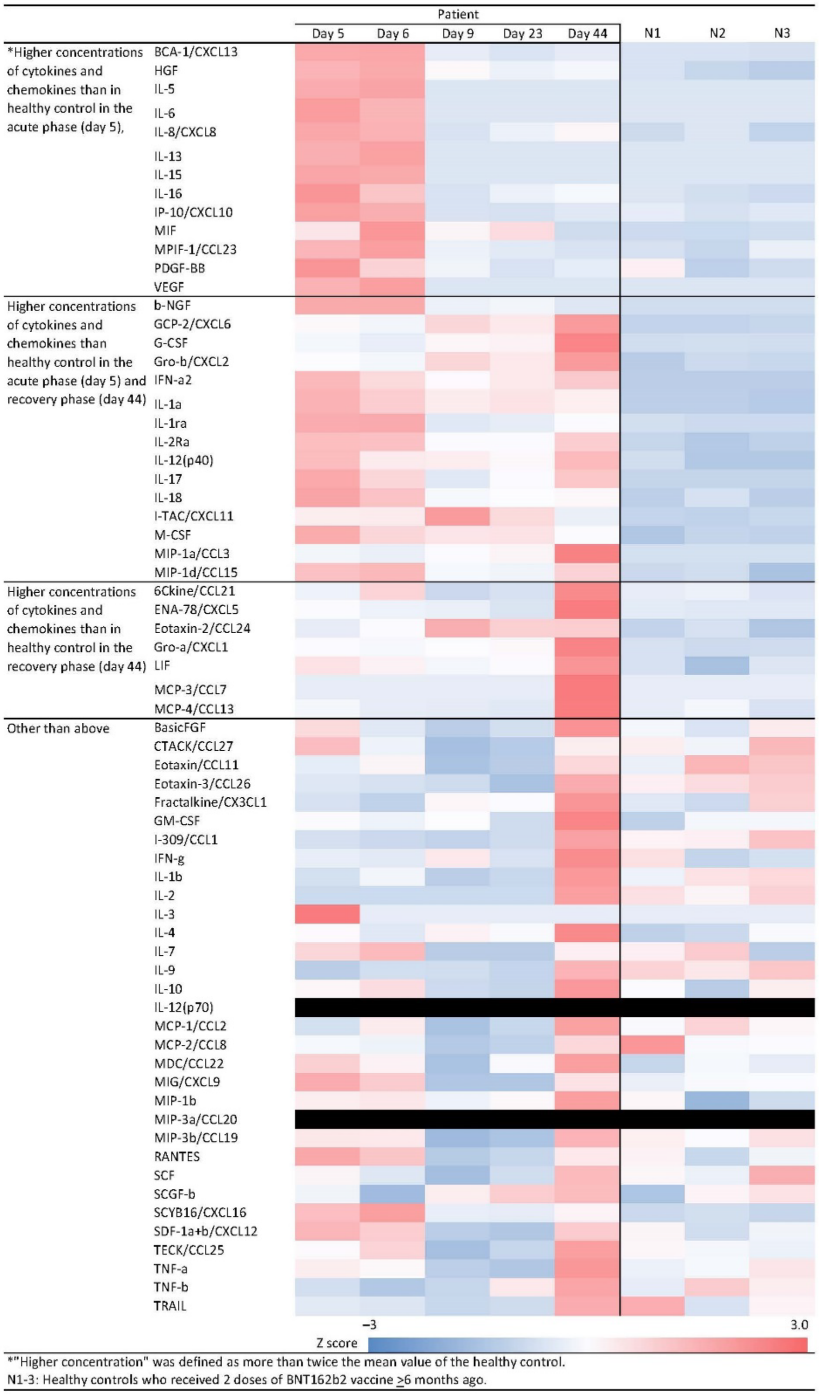

Appendix Figure 2. Profile of 67 cytokines and chemokines from a patient with multisystem inflammatory syndrome, compared with those of 3 healthy persons as controls. The controls were infection-free men 34–41 years of age who had received 2 doses of the BNT162b2 vaccine (Pfizer-BioNTech, https://www.pfizer.com).
